# Analysis of Completed Suicides in Cantabria (Spain) During the COVID-19 Pandemic: Relationship with Psychiatric Diagnoses

**DOI:** 10.1192/j.eurpsy.2026.12078

**Published:** 2026-07-31

**Authors:** A. Diusekova, A. I. De Santiago-Diaz, R. Ayesa-Arriola, C. I. Ortega-Benito

**Affiliations:** 1 Laredo regional hospital, Laredo; 2 Marqués de Valdecilla University Hospital; 3 Marqués de Valdecilla Research Institute; 4 Institute of Legal Medicine and Forensic Sciences, Santander, Spain

## Abstract

**Introduction:**

The COVID-19 pandemic in 2020 had a significant impact on mental health, intensifying risk factors associated with suicide. In Cantabria, an increase in completed suicides was observed during the third quarter of the year, although the highest number of cases among men was recorded in the first quarter.

**Objectives:**

To analyze the temporal distribution of suicides and associated psychiatric diagnoses in Cantabria during 2020 in the context of the COVID-19 pandemic.

**Methods:**

A retrospective analysis of completed suicide cases in Cantabria in 2020 was conducted (47 cases in total, 37 men and 10 women). Sociodemographic and clinical variables were examined, with particular attention to the temporal distribution of cases and the prevalence of psychiatric diagnoses among the deceased.

**Results:**

In 2020, a total of 47 completed suicides were recorded in Cantabria: 37 men and 10 women. Among men, the highest number of cases occurred in the first quarter (15 cases), followed by a peak in the third quarter (12 cases). Among women, there was a progressive increase that culminated in an absolute peak in the third quarter: 2 cases in the first quarter, 2 in the second, and 6 in the third. Regarding the presence of mental disorders, 18 men (48.6%) and 8 women (80%) had a previous psychiatric diagnosis.

**Conclusions:**

The findings show sex-related differences in the temporal distribution of suicides in Cantabria during 2020. Among men, the highest number of cases occurred in the first quarter, with an additional peak in the third quarter. Among women, there was a progressive increase, reaching its highest point in the third quarter. Most female cases, and nearly half of male cases, had a history of mental disorders. These data highlight the need to strengthen mental health resources and to implement specific prevention strategies, particularly in crisis situations such as the COVID-19 pandemic.

**Disclosure of Interest:**

None Declared

